# Some Thermomagnetic and Mechanical Properties of Amorphous Fe_75_Zr_4_Ti_3_Cu_1_B_17_ Ribbons

**DOI:** 10.3390/ma15010368

**Published:** 2022-01-04

**Authors:** Mariusz Hasiak, Jan Świerczek

**Affiliations:** 1 Department of Mechanics, Materials and Biomedical Engineering, Wrocław University of Science and Technology, Smoluchowskiego 25, 50-370 Wroclaw, Poland; 2 Department of Physics, Częstochowa University of Technology, Armii Krajowej Ave. 19, 42-200 Częstochowa, Poland; jan.swierczek@pcz.pl

**Keywords:** amorphous metallic alloys, X-ray diffraction, Mössbauer spectroscopy, magnetization, isothermal magnetic entropy change, refrigerant capacity, mechanical hardness

## Abstract

The microstructure, revealed by X-ray diffraction and transmission Mössbauer spectroscopy, magnetization versus temperature, external magnetizing field induction and mechanical hardness of the as-quenched Fe_75_Zr_4_Ti_3_Cu_1_B_17_ amorphous alloy with two refractory metals (Zr, Ti) have been measured. The X-ray diffraction is consistent with the Mössbauer spectra and is characteristic of a single-phase amorphous ferromagnet. The Curie point of the alloy is about 455 K, and the peak value of the isothermal magnetic entropy change, derived from the magnetization versus external magnetizing field induction curves, equals 1.7 J·kg^−1^·K^−1^. The refrigerant capacity of this alloy exhibits the linear dependence on the maximum magnetizing induction (*B_m_*) and reaches a value of 110 J·kg^−1^ at *B_m_* = 2 T. The average value of the instrumental hardness (HV_IT_) is about 14.5 GPa and is superior to other crystalline Fe-based metallic materials measured under the same conditions. HV_IT_ does not change drastically, and the only statistically acceptable changes are visibly proving the single-phase character of the material.

## 1. Introduction

When searching for magnetic materials, finding the most suitable one for solid refrigerant applications has posed a great challenge in the last two decades [1,2,3]. Contrary to classical refrigeration, which is based on the compression and expansion of gases, magnetic refrigeration is more environmentally friendly and more energetically efficient. It is a direct application of the magnetocaloric effect, i.e., the temperature change (∆*T_ad_*) during the adiabatic magnetization or demagnetization of a refrigerant material. ∆*T_ad_* is related to the isothermal magnetic entropy change during the isothermal magnetization process (∆*S_M_*), especially in magnetic materials exhibiting the second-order ferromagnetic–paramagnetic phase transition [1,3]. Thus, the magnetocaloric response of ferromagnetic materials can be measured directly (∆*T_ad_*) or indirectly (∆*S_M_*). The maximum ∆*S_M_* exists near the Curie point (*T_C_*) of the material. Therefore, looking for materials with a Curie temperature close to the room one is important for the application of these materials in commonly used refrigerant appliances. Transition metal-based amorphous alloys, such as soft magnetic materials, are very promising because of the ease of magnetization to saturation, the dependence of *T_C_* on the chemical composition [4], the thermal history of the specimens [5], and the low hysteresis losses and eddy current losses due to the high electric resistivity. Despite the peak values of the isothermal magnetic entropy change in Fe-based amorphous alloys being rather modest when compared with the rare-earth-based compounds [6], considerable differences in the costs of raw elements make them attractive. An enhanced magnetocaloric effect was observed in the amorphous Fe_92−x_Zr_7_B_x_Cu_1_ alloys series (x = 0–23 at. %) [7], in Fe_80-x_M_x_B_10_Zr_9_Cu_1_ (M = Ni, Ti; x = 0, 3, 5) [8] and in Fe_86−x_B_x_Mn_4_Zr_8_Nb_2_ (x = 4, 8, 12, 16 and 20 at. %) [9] multicomponent amorphous alloys. The modest peak values of ∆*S_M_* are usually accompanied by a broad full width at a half maximum in the materials, showing the second-order ferromagnetic–paramagnetic phase transition, resulting in a large refrigerant capacity (RC). NANOPERM-type Fe-M-(Cu)-B (M = Nb, Zr, Mo, Hf or Ti) amorphous alloys have also been studied under the aspect of applying them as excellent soft magnetic materials [10]. They exhibit two well-separated stages of crystallization which enable them to obtain nanocrystalline materials by conventional annealing [10]. Heat treatments at temperatures close to or above the onset of primary crystallization lead to the formation of a α-Fe granular phase embedded in the residual amorphous matrix. If the volume fraction of the crystalline phase is equal to about 0.6–0.7, the composite material shows excellent soft magnetic properties at temperatures lower than the Curie point of the intergranular amorphous phase. The magnetic softening is due to the averaging out of the magnetocrystalline anisotropy [11,12]. Additionally, if the volume fraction of the crystalline phase is about 0.6, the effective magnetostriction becomes close to zero because of the compensation of the positive contribution from the amorphous matrix and of the negative one originating from the α-Fe crystalline phase. NANOPERM-type amorphous and nanocrystalline materials containing one refractory element (Nb, Zr, Mo or Hf) have a huge representation in the literature [10]. NANOPERM-type nanocrystalline alloys are composites containing different magnetic phases. Each of them contributes to the effective magnetic entropy change. Generally, ∆*S_M_* in nanocrystalline materials is lower than in the corresponding amorphous precursors [13]. Thus, from an application point of view, as solid refrigerants, amorphous alloys in the as-quenched state and after annealing within the amorphous state are more interesting than nanocrystalline composites originating from them.

As mentioned above, the Curie temperature of the amorphous alloys can be easily tuned by the addition of refractory metals to Fe-B amorphous systems. It is reported [14,15] that *T_C_* in the Fe_80_M_7_Cu_1_B_12_ amorphous alloys depends on the composition and equals 265, 333 and 413 K for M = Mo, Nb and Ti, respectively. It is known that Curie points in amorphous alloys can be modified not only by changes in the chemical composition but by proper heat treatments within the amorphous state of a precursor, as well [16]. Such facts are very important if the application of these materials as refrigerants is considered [16,17]. It is also very interesting to combine magnetic properties with mechanical ones. In this paper, the microstructure and some thermomagnetic and mechanical properties of the multicomponent, rapidly quenched Fe_75_Zr_4_Ti_3_Cu_1_B_17_ amorphous alloy in the as-quenched state are studied. The alloy contains two refractory elements (metals): Zr and Ti, with different atomic radii of 0.155 and 0.140 nm in Zr and Ti, respectively [18]. As a comparison, the atomic radius of the Fe atom is 0.140 nm [18]. The presence of two refractory metals with different atomic radii improves the glass-forming ability of the Fe-B amorphous system. Moreover, nonmagnetic zirconium and titanium atoms affect its magnetization and Curie temperature. In NANOPERM-type amorphous alloys, the refractory elements’ content does not exceed 10 at. %, so as not to distinctly diminish their magnetization. Additionally, due to atomic radii, Mo, Nb, Zr and Ti lower the exchange interactions between magnetic atoms (Fe-Fe) and indirectly lower the Curie temperature. Under this aspect, Ti seems to be less effective [14,15]. We decide to combine Zr and Ti to obtain a wider spectrum of exchange interactions in Fe-Fe pairs. The microstructure of the alloy is revealed by X-ray diffraction and transmission Mössbauer spectroscopy. The microstructure studies are accompanied by magnetization versus temperature, magnetizing field induction and mechanical hardness measurements. The magnetic entropy change and refrigerant capacity were computed from the isothermal magnetization versus applied field induction curves.

## 2. Materials and Methods

The amorphous ribbons, 10 mm wide and 20 μm thick, with the composition Fe_75_Zr_4_Ti_3_Cu_1_B_17_ were obtained by rapid quenching on a single copper roller. The differential scanning calorimetry (DSC) curve at the heating rate of 10 K/min was obtained by a NETZSCH STA 449F1 (NETZSCH-Gerätebau GmbH, Selb, Germany) set-up. The structure of ribbons was studied by X-ray diffraction and transmission Mössbauer spectroscopy. The shiny surfaces of the ribbons were exposed to X-rays, and diffraction patterns in the 2θ range of 30–110° were recorded at the ambient temperature by a Bruker-AXS, type D8 Advanced X-ray diffractometer (Bruker AXS Gmbh, Karlsruhe, Baden-Wurtemberg, Germany). Transmission Mössbauer spectra were recorded at room temperature (300 K) by a conventional constant acceleration spectrometer with a ^57^Co(Rh) radioactive source. The spectrometer was calibrated and the isomer shift was given with respect to α-Fe polycrystalline foil. Spectra fittings were performed using the Normos package according to the procedure described in [19]. The specific magnetization, *M* (magnetic moment per unit mass), versus the temperature in the 300–550 K range at the magnetizing field induction of *B* = μ_0_*H* = 5 mT, 10 mT and 50 mT (where μ_0_ is the vacuum permeability and *H* is the magnetizing field strength) was measured for samples in the form of a strip 8 mm long and 1 mm wide by a VersaLab (Quantum Design, San Diego, CA, USA) system in zero field cooling mode. In order to reduce the demagnetization effect, apart from the sample’s shape, the magnetizing field was applied parallel to the ribbon edge in its plane. The isothermal magnetization curves, *M*(μ_0_*H*), were obtained at a 405–500 K temperature with a step of ∆*T* = 5 K and 0–2 T magnetizing field induction ranges. To determine the instrumental hardness of the as-quenched Fe_75_Zr_4_Ti_3_Cu_1_B_17_ alloy, the nanoindentation technique, with respect to the Oliver–Pharr procedure [20], was applied. 25 × 25 tests that covered an area of 360 μm × 360 μm were carried out with a maximum load of 100 mN for each specimen. A statistical analysis of the 625 obtained results was performed to show the distribution of instrumental hardness in the investigated alloy. All measurements were performed for samples in the as-quenched state.

## 3. Results and Discussion

**Figure 1:**
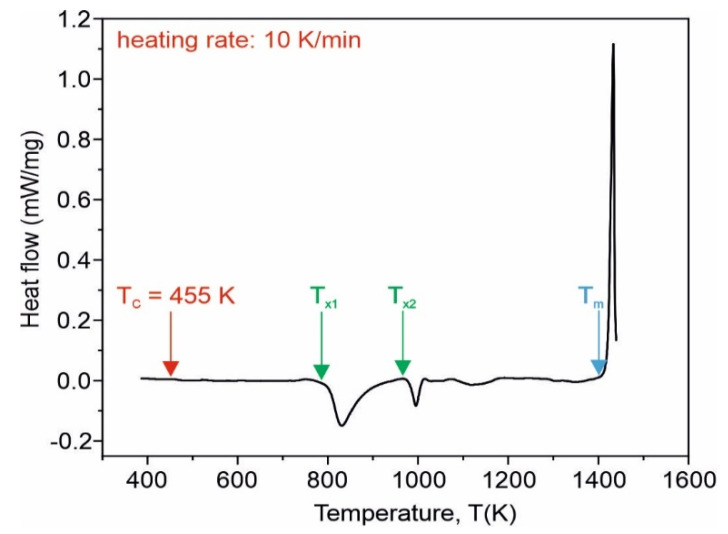
DSC curve for the as-quenched Fe_75_Zr_4_Ti_3_Cu_1_B_17_ amorphous alloy; the red arrow denotes the Curie temperature, the green arrows depict the onsets of primary and secondary crystallization temperatures *T_x1_* and *T_x2_*, and the blue arrow depicts the melting point *T_m_*.

In Figure 1, the DSC curve for the as-quenched Fe_75_Zr_4_Ti_3_Cu_1_B_17_ alloy recorded at the heating rate of 10 K/min is depicted. Two well-separated exothermic dips corresponding to the crystallization are visible. The onsets of the primary crystallization at *T_x1_* = 798 K and of the secondary one at *T_x2_* = 973 K are expected. The Curie temperature occurs at *T_C_* = 455 K and the melting point at about *T_m_* = 1409 K.

**Figure 2:**
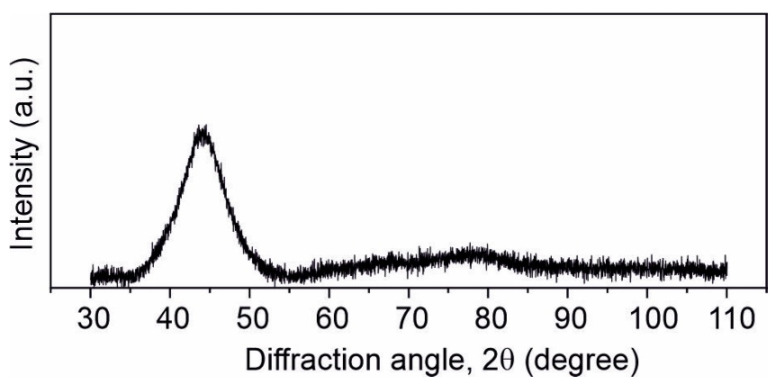
Diffraction pattern of the as-quenched Fe_75_Zr_4_Ti_3_Cu_1_B_17_ amorphous ribbon.

The X-ray diffraction pattern for the as-quenched sample, i.e., the X-ray intensity related to the maximum of the main peak versus the diffraction angle 2θ, is presented in Figure 2. The shiny surface is exposed to radiation. The penetration depth of the X-rays is about half of the ribbon thickness, like in the amorphous Fe_79_Mo_8_Cu_1_B_12_ ribbons [21], so we can assume that the pattern gives the structure information that is representative of the sample. The pattern is typical of an amorphous structure, with the main broad hump located at about 2θ = 45° and a much lower but broader one situated at about 2θ = 80°. Similar profiles of *X*-ray diffraction patterns were observed for amorphous Fe_74−x_Cr_x_Cu_1_Nb_3_Si_15.5_B_6.5_ (x = 2, 8, 10, 12, 13, 14 and 20 at. %) [22], although in some cases no broad maximum occurs at about 80° [23].

**Figure 3:**
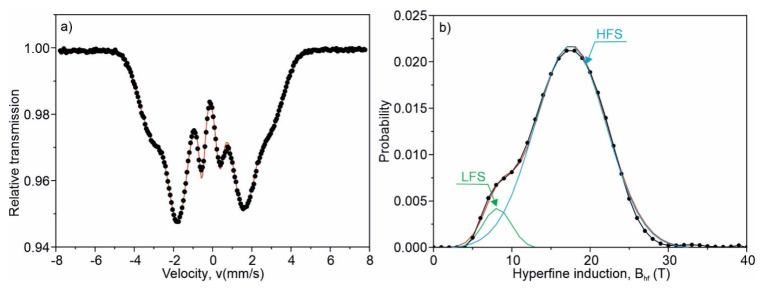
Transmission Mössbauer spectrum (**a**) and corresponding hyperfine magnetic induction distribution (**b**) of the amorphous Fe_75_Zr_4_Ti_3_Cu_1_B_17_ alloy in the as-quenched state. Decomposition of the hyperfine magnetic induction distribution into two Gaussians related to the low field sites (LFS) and high field sites (HFS) of Fe atoms.

The amorphicity of the alloy is also confirmed by Mössbauer spectroscopy. In Figure 3a, the transmission Mössbauer spectrum for the as-quenched Fe_75_Zr_4_Ti_3_Cu_1_B_17_ ribbons, recorded at the ambient temperature, is depicted. In the as-quenched state, the transmission Mössbauer spectrum is typical of amorphous ferromagnets, with broad and overlapping lines (Figure 3a). Its asymmetry results from the correlation between the hyperfine field induction (*B_hf_*) and the isomer shift (*IS*). The spectrum was fitted with a set of 40 sextets, with *B_hf_* in the range of 0–39 T changing with the step of 1 T. Taking into account the linear *IS*(*B_hf_*) relation, the corresponding distribution of the hyperfine magnetic induction at the ^57^Fe nuclei, *P*(*B_hf_*), is in this case obtained and shown in Figure 3b. Some best-fitted hyperfine parameters are listed in Table 1. The bimodal character of the *P*(*B_hf_*) distribution is visible. *P*(*B_hf_*) can be presented as the sum of two Gaussian distributions centered at 8.2 T and 17.5 T. These Gaussian distributions correspond to low (LFS) and high (HFS) field sites of Fe atoms with two different topological and chemical short-range orderings. Roughly speaking, the Fe sites possessing all Fe atoms as nearest neighbors correspond to LFS (clusters) [24], whereas Fe sites with B, Zr, Ti or Cu atoms in the nearest neighborhood are related to HFS. It is worth noticing that the probability of the paramagnetic spectrum component (*B_hf_* = 0) is equal to zero (Figure 3b) in the as-quenched state.

**Figure 4:**
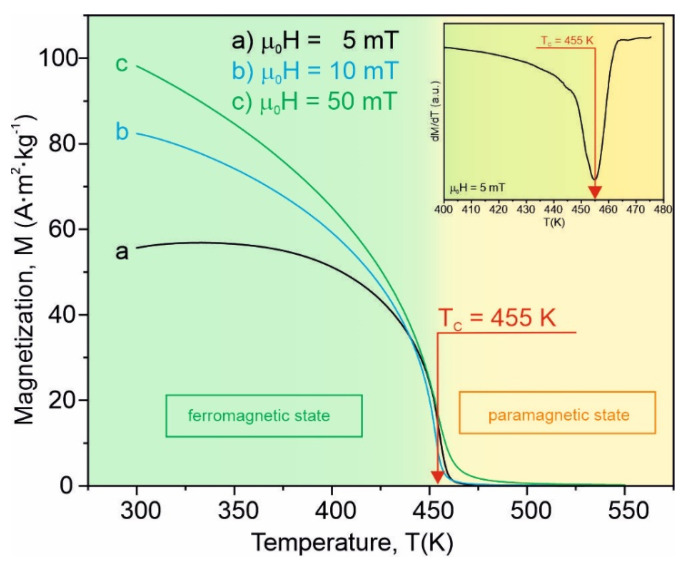
The specific magnetization *M* as a function of the temperature *T* for the amorphous Fe_75_Zr_4_Ti_3_Cu_1_B_17_ alloy in the as-quenched state at a magnetizing field induction of (**a**) μ_0_*H* = 5 mT, (**b**) μ_0_*H* = 10 mT and (**c**) μ_0_*H* = 50 mT. As an inset, to determine the Curie point, the derivative ∂M/∂T versus the temperature curve is shown.

According to Figure 2 and Figure 3, the amorphous Fe_75_Zr_4_Ti_3_Cu_1_B_17_ alloy can be treated as a single phase. The magnetization, *M*, versus the temperature in the 300–550 K range, measured at a constant external magnetizing field of *B* = μ_0_*H* = 5 mT, 10 mT and 50 mT for the as-quenched specimens, is shown in Figure 4. In the case of μ_0_*H* = 5 mT, the magnetization increases slightly with the temperature before a critical region is reached and a drop of *M* occurs. To elucidate such a behavior, the different temperature dependences of the effective magnetic anisotropy field and the magnetization should be taken into account [25]. The effective anisotropy constant decreases faster with temperature than the magnetization, and at a constant external magnetizing field an increase of *M* versus temperature may be observed, providing that the anisotropy field is higher or comparable with the external one. When the external magnetizing field exceeds the anisotropy field, a monotonical decrease of the magnetization may occur in a similar way in the cases of *B* = μ_0_*H* = 10 mT and 50 mT.

**Figure 5:**
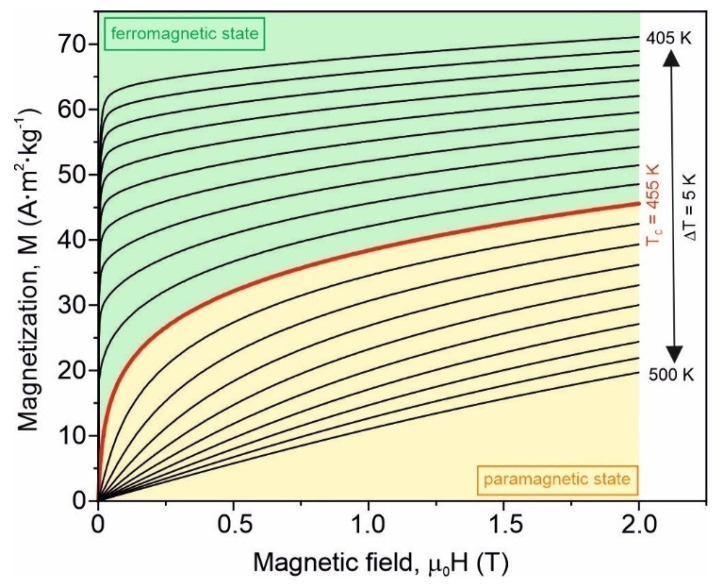
Set of isothermal magnetization curves in the temperature range 405–500 K with a step of ∆*T* = 5 K and at a maximum magnetizing field induction of *B* = μ_0_*H* = 2 T for the amorphous Fe_75_Zr_4_Ti_3_Cu_1_B_17_ alloy.

**Figure 6:**
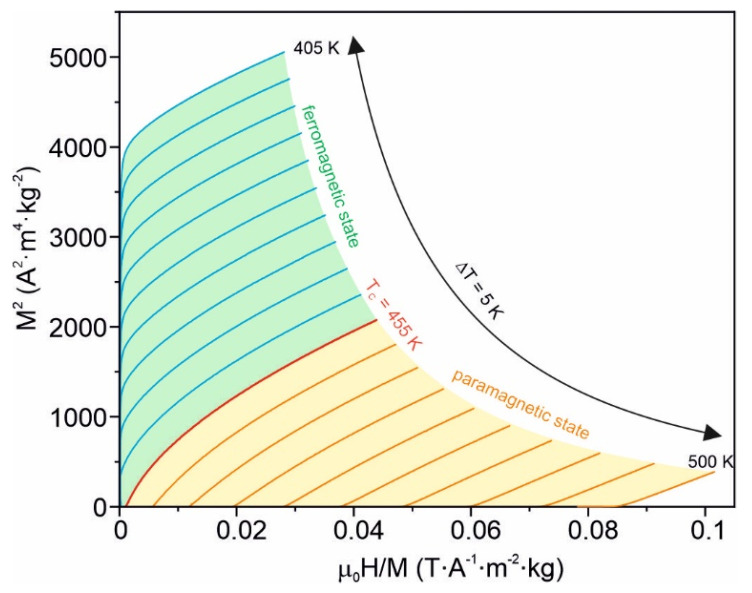
Arrott plots, i.e., *M*^2^ as a function of μ_0_*H/M* for the amorphous Fe_75_Zr_4_Ti_3_Cu_1_B_17_ alloy.

The derivative ∂M/∂T with the fast Fourier transformation smoothing for the as-quenched samples is depicted as an inset in Figure 4. The minimum of the ∂M/∂T curve corresponds to the Curie temperature of the amorphous Fe_75_Zr_4_Ti_3_Cu_1_B_17_ alloy which is equal to *T_C_* = (455 ± 2) K. The Curie point of the amorphous Fe_83_B_17_ alloy is about 593 K [26]. In accordance with the Bethe–Slater curve, the exchange interaction is very sensitive to the distance between magnetic moments [10]. If Ti and Zr atoms are situated between Fe ones, they enlarge the distance between the magnetic atoms, leading to a decrease of the exchange interaction. Such an effect is more enhanced in the case of the Zr atom owing to its atomic radius. The wide spectrum of the exchange interaction strength gives a rather high Curie temperature. The family of isothermal magnetization curves *M*(μ_0_*H*) in the temperature range 405–500 K at a maximum magnetizing field induction of 2 T for this alloy is presented in Figure 5. The Arrott plots, i.e., *M*^2^ as a function of μ_0_*H/M* in the abovementioned temperature and magnetizing field induction ranges, are presented in Figure 6.

**Figure 7:**
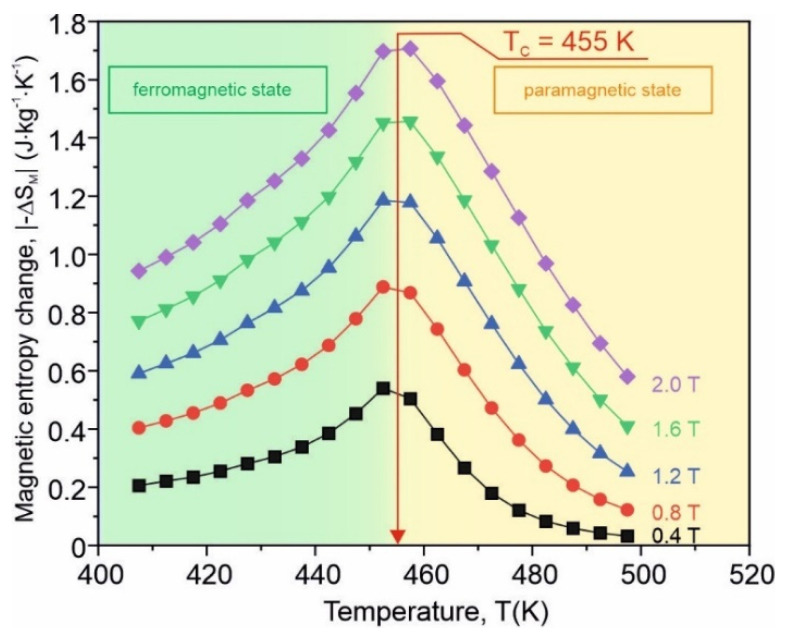
The isothermal magnetic entropy change versus temperature at six different values of the external magnetizing field induction in the as-quenched amorphous Fe_75_Zr_4_Ti_3_Cu_1_B_17_ alloy.

The positive slope of the Arrott plots, according to the Banerjee criteria [27], confirms the second-order ferromagnetic–paramagnetic phase transition in the investigated alloy, and hence the isothermal magnetic entropy change can be obtained from one of the Maxwell thermodynamic equations [3]:

(1) $$\Delta S_{M}=\int_{0}^{B_{m}}{\left(\frac{\partial M\left(T,B\right)}{\partial T}\right)}_{B}dB$$

where *B_m_* denotes the maximum magnetizing field induction and ∂ the partial derivative. ∆*S_M_* is computed using the numerical approximation described in detail in [28]. In Figure 7, the isothermal magnetic entropy change versus the temperature for five different values of the maximum magnetizing field induction is presented. It is seen that ∆*S_M_* reaches its maximum near the Curie point of the investigated material. Its peak value at *B_m_* = 2 T is about 1.7 J·kg^−1^·K^−1^. Moreover, ∆*S_M_* depends on *B_m_* according to the relation [29,30]:

(2) $$\Delta S_{M}=CB_{m}^{n}$$

where *C* is temperature-dependent and *n* depends on the magnetic state of the sample. The exponent *n* can then be obtained from the equation:

(3) $$\ln\left|\Delta S_{M}\right|=\ln\left|C\left(T\right)\right|+n\cdot \ln B_{m}$$

**Figure 8:**
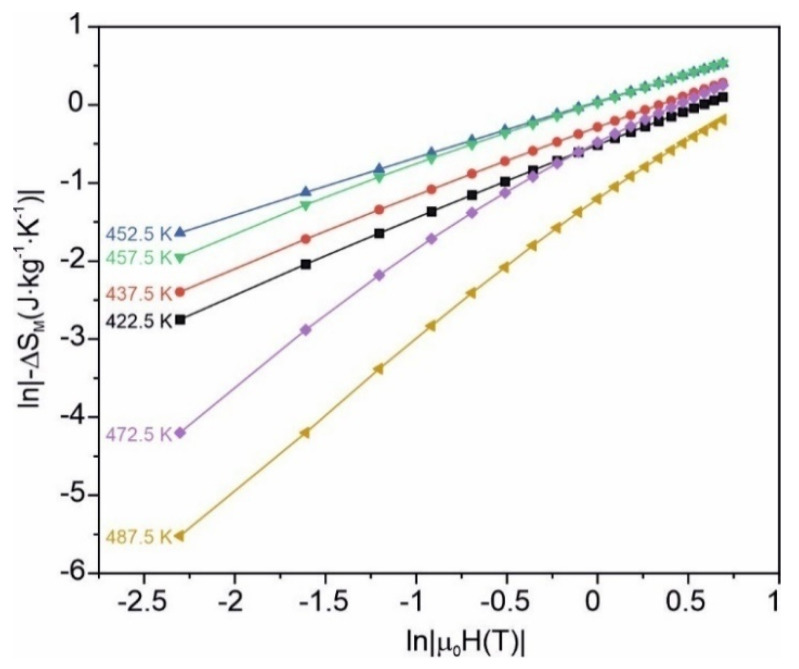
ln|∆*S_m_*| as a function of ln*B_m_* for the as-quenched amorphous Fe_75_Zr_4_Ti_3_Cu_1_B_17_ alloy.

The relation (3) for six chosen temperatures is depicted in Figure 8. For some temperatures, the discrepancy from the linearity is visible.

**Figure 9:**
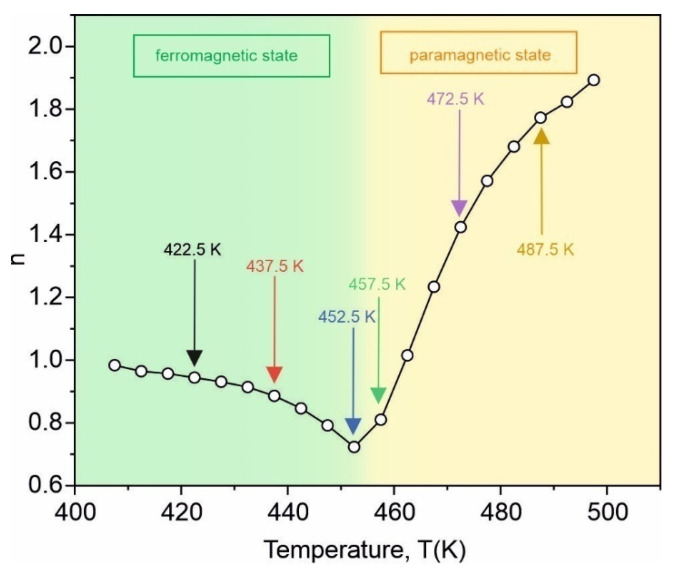
Exponent *n* versus temperature for the as-quenched amorphous Fe_75_Zr_4_Ti_3_Cu_1_B_17_ alloy.

The exponent *n* directly derived from the linear part of the relation (3) plotted versus the temperature is presented in Figure 9. The exponent is about 1 at a low temperature in the ferromagnetic state, reaches its minimum at a temperature close to *T_C_* and does not amount to 2 in the paramagnetic state, although the tendency to increase is observed. For a single-phase material exhibiting the second-order magnetic phase transition, three characteristic values of n have been reported: *n* = 1 at a temperature below the Curie point of the material, $n=1+\frac{1}{\delta }\left(1-\frac{1}{\beta }\right)$ at *T_C_*, where *δ* and *β* are critical exponents, and *n* = 2 above *T_C_* if the material fulfills the Curie–Weiss law [29].

**Figure 10:**
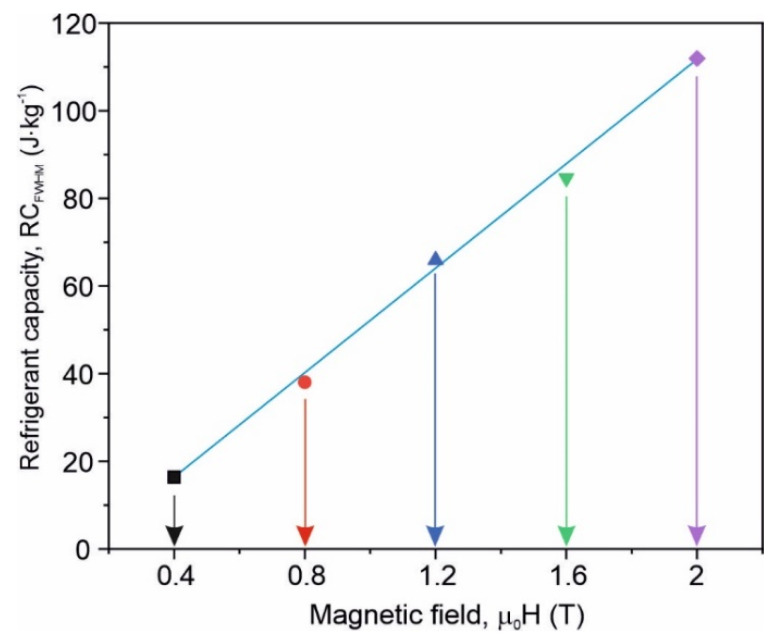
Refrigerant capacity, RC, versus maximum magnetizing field induction μ_0_*H* for the as-quenched amorphous Fe_75_Zr_4_Ti_3_Cu_1_B_17_ alloy.

Thus, one can say that the investigated material can be treated as a single phase well below and well above the Curie point. As can be seen from Figure 7, the ∆*S_M_*(*T*) curves are rather broad, and taking into account the application point of view, the refrigerant capacity (RC) estimated as a product of the ∆*S_M_* peak value and temperature span at the half value of the magnetic entropy change should be considered. In Figure 10, RC versus the maximum magnetizing field induction for the as-quenched amorphous Fe_75_Zr_4_Ti_3_Cu_1_B_17_ alloy is depicted. The almost linear dependence is visible. The refrigerant capacity value at *B_m_* = 2 T (about 110 J·kg^−1^) is typical for magnetic amorphous refrigerants [3].

In Table 2, the Curie points and the peak values of ∆*S_M_* obtained at the maximum magnetizing field of 2 T for some Fe-based amorphous alloys in the as-quenched state are listed. It can be seen that the higher the Curie point, the larger the maximum entropy change. Such empirical behavior has been observed in transition metals-based amorphous alloys [17] and has not been fully understood yet. An attempt to elucidate this behavior qualitatively has been undertaken in [31]. The exchange interactions between magnetic moments in amorphous alloys are distributed in strength and sign, leading to different magnetic moment configurations: from random via noncollinear to simply collinear [32]. *T_C_* is also correlated with the exchange interaction distributions, and one can believe that the higher the Curie point is, the more a collinear magnetic moment configuration occurs. The magnetic entropy is the measure of the magnetic moment disorder. Its change is larger during the transition from a more collinear (ferromagnetic) state to a random (paramagnetic) one [29]. One can say that because of the rather high Curie temperature, the investigated alloy is less attractive as a solid state refrigerant material in commonly used cooling appliances. In the future, it might be used for special applications, for example in the spacecraft industry.

**Figure 11:**
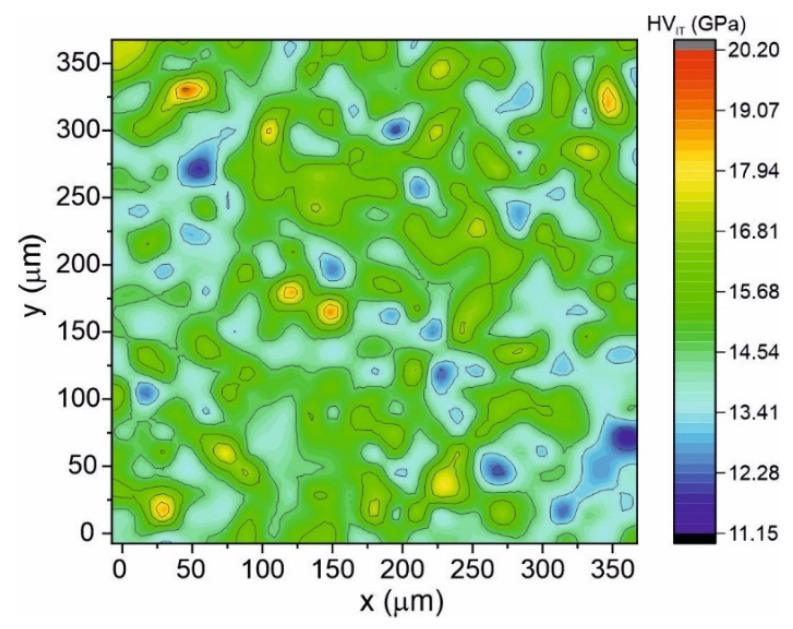
2D map distribution of the instrumental hardness HV_IT_ plotted for 25 × 25 indents recorded for the as-quenched amorphous Fe_75_Zr_4_Ti_3_Cu_1_B_17_ alloy.

The mechanical properties of the studied alloy also determine its potential application. Figure 11 shows the distribution of the instrumental hardness (HV_IT_) measured for an area of 360 μm × 360 μm. One can see that the instrumental hardness does not drastically change and that only statistically acceptable changes are visible. The statistically allowed fluctuations of HV_IT_ values are related to the nonuniform internal structure within the amorphous phase and stresses introduced to the sample during the rapid cooling production process.

**Figure 12:**
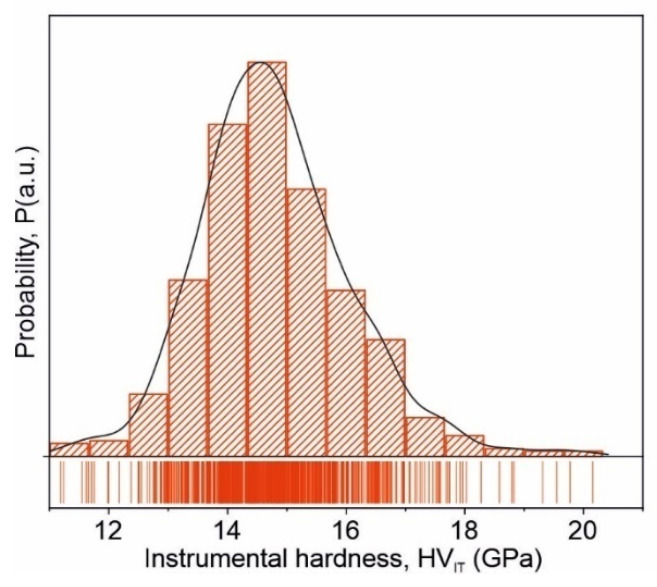
Histogram of the instrumental hardness P(HV_IT_) constructed for 625 indents recorded for the as-quenched amorphous Fe_75_Zr_4_Ti_3_Cu_1_B_17_ alloy.

A more detailed statistical study of the hardness distribution is presented in Figure 12. The single modal distribution with an average value of HV_IT_ = 14.5 GPa characterizes the mechanical properties of the investigated ribbons, as well as emphasizing the single-phased nature of the alloy. The enhanced instrumental hardness value of the investigated alloy, when compared with typical crystalline alloys (e.g., HV_IT_ = 3.7 GPa for 316L stainless steel and HV_IT_ = 6 GPa for Ti6Al4V alloy [34]), results from its amorphous structure. Based on the obtained results, it can be stated that the microstructure, in addition to the chemical composition, significantly affects the mechanical properties of metallic materials.

## 4. Conclusions

The NANOPERM-type amorphous Fe_75_Zr_4_Ti_3_Cu_1_B_17_ alloy with two refractory metals (Zr and Ti) seems to be almost single-phase, as revealed by X-ray diffraction and transmission Mössbauer spectroscopy. The Curie point of the alloy is about 455 K, and the maximum magnetic entropy change at *B_m_* = 2 T equals 1.7 J·kg^−1^·K^−1^. In comparison with other Fe-based amorphous alloys with *T_C_* near room temperature, the investigated alloy obeys the empirical rule: the higher the Curie temperature, the larger the peak value of ∆*S_M_*. The average value of HV_IT_ is about 14.5 GPa, which is superior to other crystalline Fe-based metallic materials. The instrumental hardness exhibits no sudden changes; only statistically acceptable ones are visible, confirming the single-phase character of the alloy.

## Figures and Tables

**Table 1:** Some best-fitted hyperfine parameters of the transmission Mössbauer spectrum for the amorphous Fe_75_Zr_4_Ti_3_Cu_1_B_17_ alloy in the as-quenched state: $\bar{B_{hf}}$—the average value of the hyperfine field induction, Δ$B_{hf}$—its standard deviation, $\bar{IS}$—the average value of the isomer shift, $A_{2,5}$—the relative intensity of the second and fifth line, and A—the relative area of the spectrum. Statistical uncertainties for the last significant figure are given in brackets.

Thermal History of the Sample	Subspectra	$\bar{B_{hf}}\;(T)$	$\Delta B_{hf}\;(T)$	$\bar{IS}\;(\mathrm{mm}/s)$	$A_{2,5}$	A (%)
as-quenched	set of sextets	16.7 (1)	5.0 (1)	−0.090 ± 0.003	2.93 (2)	100

**Table 2:** Curie temperature, *T_C_*, and peak entropy change, |∆*S_Mpeak_*|, at *B_m_* = 2 T for some chosen transition metals-based amorphous alloys in the as-quenched state.

Composition	*T_C_* (K)	|∆*S_Mpeak_*| (J·kg^−1^·K^−1^)	Reference
Fe_76_Mo_10_Cu_1_B_13_	277	0.88	[5]
Fe_70_Mn_10_Mo_5_B_15_	298	0.89	[33]
Fe_69.75_Co_0.25_Mn_10_Mo_5_B_15_	320	0.92	[33]
Fe_69.5_Co_0.5_Mn_10_Mo_5_B_15_	370	1.30	[33]
Fe_75_Zr_4_Ti_3_Cu_1_B_17_	455	1.70	[This work]

## Data Availability

Data are included in the article.
